# Predictors of seeking financial compensation following motor vehicle trauma: inception cohort with moderate to severe musculoskeletal injuries

**DOI:** 10.1186/s12891-017-1535-z

**Published:** 2017-05-02

**Authors:** Darnel Murgatroyd, Ian A. Harris, Jian Sheng Chen, Sam Adie, Rajat Mittal, Ian D. Cameron

**Affiliations:** 1 John Walsh Centre for Rehabilitation Research, The University of Sydney, Kolling Institute, Sydney, NSW Australia; 2 Orthopaedic Surgery, Ingham Institute for Applied Medical Research, South Western Sydney Clinical School, UNSW, Sydney, NSW Australia; 3 Department of Rheumatology, Royal North Shore Hospital, St Leonards, NSW Australia; 4 South West Sydney Clinical School, UNSW, Sydney, NSW Australia; 5 Ingham Institute for Applied Medical Research, South Western Sydney Clinical School, UNSW, Sydney, NSW Australia; 6 Rehabilitation Medicine, John Walsh Centre for Rehabilitation Research, The University of Sydney, Kolling Institute, Sydney, NSW Australia

**Keywords:** Compensation and redress, Wounds and injury, Multiple trauma

## Abstract

**Background:**

Compensation related factors have been repeatedly associated with poor recovery following orthopaedic trauma. There is limited research into the factors associated with seeking financial compensation. Further understanding of these factors could facilitate injury recovery by purposeful compensation scheme design. The aim of this study was to investigate the predictors of seeking financial compensation, namely making a claim and seeking legal representation, following motor vehicle related orthopaedic trauma. The study was conducted in New South Wales (NSW), Australia, in motor vehicle crash and workers’ compensation schemes.

**Methods:**

Participants were patients admitted with upper or lower extremity factures following a motor vehicle crash to two trauma hospitals. Data were collected at baseline within two weeks of injury. Participants were followed up at six months. Analysis involved: descriptive statistics for baseline characteristics; comparison of compensable and non-compensable participants with Analysis of Variance (ANOVA) and chi-squared tests; and logistic regression for predictor models.

**Results:**

The cohort consisted of 452 participants with a mean age 40 years; 75% male; 74% working pre-injury; 30% in excellent pre-injury health; 56% sustained serious injuries with an Injury Severity Score (ISS) 9-15; 61% had a low-middle range household income; and 35% self-reported at fault in the crash. There was no significant difference in pre-injury/baseline health between compensable and non-compensable participants. Follow up data was available for 301 (67%) participants.

The significant predictor of claiming compensation in the adjusted analysis was higher body mass index (BMI) (overweight Odds Ratio [OR] 3.05, 95% Confidence Interval [CI] 1.63-5.68; obese OR 1.63, 95% CI 0.83-3.20). Participants less likely to claim were: involved in a motorcycle crash (OR 0.47, 95% CI 0.28-0.82); socioeconomically less disadvantaged (OR 0.37, 95% CI 0.17-0.82) or least disadvantaged (OR 0.39, 95% CI 0.17-0.90); at risk for short term harm (injury) due to alcohol consumption (OR 0.56, 95% CI 0.32-0.97); and with fair-poor pre-injury health (OR 0.30, 95% CI 0.09-0.94). The predictors for seeking legal representation were speaking a language other than English at home (OR 2.80, 95% CI 1.2-6.52) and lower household income (OR 3.63, 95% CI 1.22-10.72). Participants less likely to seek legal representation were least socioeconomically disadvantaged (OR 0.15, 95% CI 0.04-0.50).

**Conclusions:**

Seeking financial compensation was associated with a higher pre-injury BMI rather than injury-related factors. Seeking legal representation was solely related to socio-economic factors.

## Background

Motor vehicle related orthopaedic trauma contributes significantly to the burden of disease and injury [1, 2] and can occur within a compensation environment providing injured people with access to financial entitlements. Compensation related factors have been linked to poor recovery after orthopaedic trauma across different compensation systems; such factors include making a claim and legal representation [3–9]. However, these negative associations have been criticised due to the potential for reverse causality bias between compensation related factors and pre-injury and/or baseline health status [10, 11]. Reverse causality is when the direction of cause and effect is contrary to what is presumed or is a two-way causal relationship [12]. For example, does poor health lead people to claim compensation or does claiming compensation cause poor health? As part of our analysis, we addressed the first part of this question.

To the authors’ knowledge two studies have compared health status between compensable and non-compensable participants and found a difference at baseline and follow up in a trauma cohort [3, 13]. Involvement in the compensation process and the stressfulness of having a claim has been associated with poorer mental health status following injury but a significant portion of that poorer status was present at baseline [14, 15]. Furthermore, baseline health is a known predictor of injury recovery [4–6].

In addition, others have pointed out the need for further comparative research between and within different jurisdictions to tease apart the complex issues surrounding compensation systems including scheme design and the societal framework in which they operate [16, 17]. Although many studies have explored the association between compensation related factors and trauma recovery, few have investigated the drivers for making a claim or seeking legal representation following motor vehicle related orthopaedic trauma.

Many compensation schemes tend to have eligibility requirements such as only being entitled to claim if they were not at fault (a fault-based scheme) or in a work-related incident regardless of fault (a no fault scheme). These requirements dictate access to financial entitlements (benefits). Recent Australian reports show that, despite being eligible, some people choose not to make a claim. The reasons are diverse, for example: a lack of awareness of eligibility, sustaining a minor injury, prolonged injury recovery, and current and/or future employment concerns [18–20]. However, that research has been focussed on work-related injuries or Whiplash Associated Disorders (WAD).

The intricate relationship between health, psychosocial and socio-economic factors, and compensation systems has been more closely examined in the qualitative literature. Researchers have looked at the impact of the claims process, interactions between injured workers, health care providers and insurers [21–23], and financial and employment considerations [24]. Results showed having a compensation claim had largely negative influence on injury recovery. However, these relationships were mostly explored during the claims process, not prior to making a claim. Background factors (i.e. those present prior to injury) may be determinants of making a claim and/or post-injury outcomes.

Hence, the study aim was to explore the predictors for seeking financial compensation, namely making a claim and seeking legal representation, following motor vehicle related orthopaedic trauma.

## Methods

### Study design

Participants were recruited for this inception cohort study from two trauma hospitals (Liverpool and St George) in Sydney, NSW, Australia from November 2007 to February 2011. The selected hospitals provided a representative sample of motor vehicle related orthopaedic trauma in NSW that required inpatient hospitalisation in NSW. Participants were followed up at 6, 12 and 24 months post injury.

Eligible patients identified via a hospital trauma database of orthopaedic admissions were invited to participate. Informed consent was obtained. Inclusion criteria were: admission to hospital within 2 weeks of injury; involvement in a motor vehicle crash; age 18 years or over; and an upper or lower extremity fracture (humerus, radius, ulna, pelvis, acetabulum, femur, patella, tibia, fibula, talus, calcaneus). An English speaking family member was used to interview eligible patients from Culturally and Linguistically Diverse (CALD) backgrounds. Patients were excluded if they had: dementia or a significant pre-existing cognitive impairment preventing the ability to consent; spinal cord injury; Glasgow Coma Score (GCS) less than 12 on admission; amputation of a limb; or isolated phalangeal, carpal, metacarpal, tarsal or metatarsal fractures.

There were 32 variables measured for each participant. Calculations for the sample size of 450 were based on an allowance of 10 participants per variable [25] and accommodated a 25% loss to follow up with reference to similar published research [8, 26].

At six, 12 and 24 months after injury follow up questionnaires were posted to participants. By 3 weeks, if there was no response participants were contacted up to six times by telephone. Questionnaires could be completed by telephone or by mail. Participants were removed from the study if non-contactable or they declined to participate. Additional information about the study methodology is published in two separate papers investigating injury recovery and return to work in the same cohort [27, 28].

Baseline data were collected in hospital within 2 weeks post injury by written questionnaire. Demographic data including date of birth, age, gender, and injury related information were obtained from hospital records and a trauma database. Selected study factors were based on relevant research and referred to the aims and objectives of the study [6, 7, 29, 30].

### Setting

In NSW at the time of the study, the Motor Accidents Authority (MAA) was the government insurance regulator of the CTP personal injury scheme, and is a privately underwritten modified common law scheme. WorkCover was the government insurance regulator of the WC scheme, and is a publically underwritten statutory benefit scheme where private insurers manage claims on behalf of WorkCover [31, 32]. In 2015, the scheme regulators amalgamated and formed the State Insurance Regulatory Authority (SIRA).

All motor vehicles travelling on public roads must be registered. To make a CTP claim a motor vehicle must also be registered and the claim is made against the driver at fault. From April 2010, anyone injured in a motor vehicle crash (regardless of fault) can access limited entitlements, that is: medical expenses and lost wages up to AUD (Australian Dollar) $5000. Before 2012, to make a WC claim a motor vehicle crash must have occurred during travel between place of employment and home, and/or any work-related place, and a person injured (regardless of fault) [31, 32].

For both schemes, a claim must be lodged within six months of injury and insurers have 3 months to determine final liability (accept/deny the claim). Provisional liability facilitates access to treatment by earlier payment of medical expenses and for WC weekly wage benefits. In WC, within 48 h insurers must be informed of an injury [32]. Entitlements include past and future losses: for example, medical expenses, loss of income, and pain and suffering/impairment. In both schemes, payments for medical expenses are made as incurred. In CTP, loss of income (including past and future), future medical expenses, and permanent impairment are paid as a lump sum at claim settlement. In WC, loss of income is paid weekly and can be lifetime depending on the level of impairment. Future medical expenses can be lifetime, and permanent impairment is paid as a lump sum. In addition, legal representation can also be sought at any time for either scheme. [31, 32]

### Injury related study factors

The Abbreviated Injury Scale (AIS) (1990 Revision, Update 98) was used to code all injuries [33]. The Injury Severity Score (ISS) and New Injury Severity Score (NISS) were calculated as measures of injury severity; these are considered indicators of potential mortality [34] and are the sum of the squares of the three highest AIS scores from different body regions (ISS) regardless of body region (NISS). The AIS ranks injuries to particular body regions on a scale from one to six (six is not survivable). Injuries were classified as minor–moderate (1-8), serious (9-15) or severe–critical (16-75) based on ISS/NISS scores [35].

### Socio-demographic study factors

Socio-demographic factors included age, gender, marital status, occupation, and education. Occupation was measured using the Australian Standard Classification of Occupations (ASCO), classifications were divided by skill level (e.g. managers/professionals, tradespersons, intermediate clerical and elementary related) [28, 36]. Current work status (yes/no) was asked with additional variables for full/modified duties (e.g. lifting restrictions, reduced hours) and full-time (usually working at least 35 h per week) or part-time (usually working one hour to 35 h per week) [37].

Household income was measured exclusive and inclusive of household structure to cater for any differences in income distribution. To calculate an adjusted income (inclusive of household structure), household income was divided by the sum of points, 1 for the first person aged ≥15 years, 0.5 for each additional person aged ≥15 years, and 0.3 for each person aged <15 years [38, 39].

The Index of Relative Socioeconomic Disadvantage (IRSD) summarises economic and social conditions within a particular area/postcode such as employment, fluency in English and household size [38]. The lowest score is indicative of greatest or most socioeconomic disadvantage and the highest score indicates least disadvantage. It can be used as a continuous variable or divided into quintiles.

### Health related study factors

Different health conditions measured as indicators of general health status at baseline were chronic illnesses – asthma, cancer, heart and circulatory conditions, diabetes, arthritis, osteoporosis, mental and behavioural problems, and neck and back problems/disorder/pain. The National Health Priority Areas initiative list these conditions as imposing high social and financial costs on Australian society) [40]. The classification was based on the Australian Bureau of Statistics (ABS) Health Survey which defines a long term condition as one which the patient currently has, and which has lasted or they expect to last for 6 months or more [39–41]. Body Mass Index (BMI) was calculated from the participant’s self-reported weight and height.

Other factors measured in accordance with the ABS Health Survey included: recent injuries (other than the motor vehicle crash) in the last 4 weeks that required medical intervention or were associated with a decrease in usual activities; medication use in the last 2 weeks for asthma, arthritis, osteoporosis, heart or circulatory conditions, diabetes, high sugar levels, mental wellbeing; and smoker status [39].

In previous research, associations were found between poor recovery and poor expectations for return to usual activities and work [29, 42–44]. There were few validated measures for self-efficacy and other similar constructs, therefore, two applicable measures from a large Canadian study of soft tissue injuries were used [43]. These were ‘do you expect to return to work (yes/no)’ and ‘when do you expect to return to usual activities’ (number of days).

A validated scale measured alcohol consumption with the first three questions of the Alcohol Use Disorders Identification Test: Self-Report Version (AUDIT-C) [45, 46]. The word ‘standard’ and ‘in the past year’ were added. Alcohol quantity was based on an Australian standard drink [47, 48]. The AUDIT-C questions measure number and frequency of drinking. The National Health and Medical Research Council (NHMRC) levels were used to assess risk of long term harm (alcohol related disease) and/or short term harm (alcohol related injury) due to alcohol consumption [47]. The risk levels for long term harm were low risk, risky, and high risk based on the number of standard drinks/week consumed over the past year. The risk level for short term harm was if ≥6 standard drinks were consumed on one occasion, on one day over the past year.

Because these measures did not match the AUDIT-C categories, to compare results an algorithm was calculated from the Bettering the Evaluation of Care and Health (BEACH) Survey, (Professor K Conigrave, personal communication March 19, 2007). Categories for other study factors are explained in the tables and our other published research from the same cohort that investigated predictors of injury recovery and return to work. [27, 28]

### Compensation related measures

Most compensation related factors were not recorded at baseline because the questions were unanswerable (within 2 weeks of injury). At 6 months post injury the questions asked were: claim made (yes/no), claim type (Compulsory Third Party [CTP]/Workers Compensation [WC]/other), claim accepted (yes/no/don’t know), and legal representation obtained (yes/no). Claim made ‘yes’ was defined as making a personal injury claim of any type (CTP, WC or other) to access entitlements, which included a CTP Accident Notification Form (ANF) for expenses < AUD$5000 within 28 days of injury. Self-reported fault of the driver was measured at baseline (i.e. whether the driver considered themselves to have caused the crash). Pedestrians and passengers were considered not at fault (at baseline) because road rules dictate that vehicles must give way to pedestrians. However, both have a responsibility for their own safety and where they fail to take care, rules of apportionment under contributory negligence apply, that is: the insurer believes that the person contributed to the crash and/or their injuries (e.g. not wearing a seatbelt or crossing the road at a red traffic light can result in reduced financial entitlements at settlement [49].

### Data analysis

Descriptive statistics were used to summarise baseline characteristics of the participants by claim status (i.e. made a claim Yes/No) at 6 months. The differences in the baseline characteristics between those that claimed compensation and those that did not were compared using Analysis of Variance (ANOVA) tests for continuous variables and chi-squared tests for categorical variables. The variables met the assumptions of independence, homoscedasticity and normality. Chi-squared tests were also undertaken to determine relationships between claim type and legal representation as well as claim type and claim acceptance. Logistic regression models were employed to determine predictors of claiming compensation and legal representation at six months. All potential predictor variables were considered for the final predictive models. We included variables with a *p*-value of <0.20 in univariate analyses and retained only variables with a *p*-value of ≤0.10 in the final models. Variables for the final model were selected using a backward elimination technique based on changes in likelihood ratios. The Spearman’s Rank-Order Correlation Coefficient and the Variance Inflation Factor (VIF) tests were performed to check for multicollinearity between variables in the model. The C-statistic (equivalent to the area under the Receiver Operating Characteristic curve) was used as an indication of the predictive accuracy of the final models. To assess the impact of eligibility factors, sensitivity analysis was conducted using only those participants that were eligible (*n* = 180). All data analysis was performed using SPSS statistical software version 21 (SPSS Inc, USA).

## Results

### Baseline characteristics

There were 840 eligible participants admitted to both hospitals (November 2007 - February 2011), 491 were screened with 452 (92%) consenting to participate. Due to limited resources, 349 eligible participants were not screened. Additional analysis of those eligible but not screened was not possible due to lack of patient consent ethical considerations. Potential participants were likely to have been missed at random. There were 31 refusals and eight who were discharged and unable to be contacted. Reasons for refusals were: not interested (10); language difficulties (5); and already involved in another study (1). The remaining 15 gave no reason. Further results about the cohort’s baseline characteristics, injury recovery and return to work outcomes are published in two additional papers [27, 28].

The baseline characteristics are described from a total of 452 unless otherwise stated. The mean age of participants in the study was 40 years (17.1 SD) with a range of 18-87 years. The majority 253 (56%) ISS and 189 (42%) NISS sustained serious injuries with ISS/NISS of 9-15; 124 (27%) ISS and 93 (21%) NISS sustained minor to moderate injuries (ISS/NISS) of 1-8; and the reminder 75 (17%) ISS and 170 (38%) NISS sustained severe to critical injuries (ISS/NISS) of 16-75. Male participants made up the majority at 332 (75%). More participants 279 (62%) were in the IRSD middle and lower two quintiles with corresponding middle and lower household income brackets. Seventeen percent (75) had obtained a bachelor degree or above.

At the time of injury 334/450 (74%) participants were working, mostly full time 273/330 (83%) and full duties 321/334 (96%). Expectations to return to work were high 299/333 (90%) as was job satisfaction 320/334 (96%). While only 267/424 (63%) participants expected to return to usual activities in ≤90 days. Thirty five percent (157/451) considered themselves at fault in the crash and 410 (91%) crashes occurred on a public road.

Excellent pre-injury health was perceived by 137 (30%), while 31 (7%) considered it fair to poor. Regarding other health factors, 157 (35%) reported one or more chronic illnesses, 268/448 (60%) were overweight or obese, 157 (27%) took medication within the last 2 weeks, and 125/450 (28%) were current smokers. There was a higher risk of short term harm for 254 (56%) due to alcohol consumption, that is: injury due to alcohol consumption, but a low risk of long term harm for 420/451 (93%), that is: disease due to alcohol consumption (data not shown).

### Compensation and participant status

In line with the study aims, participants were analysed by their compensation status (i.e. claim made or no claim made regardless of claim acceptance). This included all variables measured at baseline. There were 301 (67%) participants who completed the 6 month questionnaire, of those 294 responded to the compensation related questions and 61% (179/294) made a claim, of those 125/179 (70%) sought legal representation. The characteristics of each group are illustrated in an earlier publication [27], but briefly the results showed that there were significant differences between the compensable and non-compensable groups. Notably, most of those eligible to claim under NSW legislation made a claim: 82% of participants who self-reported not at fault and 95% who had a crash on a public road. There were no significant differences found in pre-injury or baseline general health status between the two groups, although the measures pertained mostly to physical health.

The significant differences at follow up between responders and non-responders have been reported previously [27]. In summary, non-responders were young and pre-injury, they were more likely to be single, have smoked, not to have worked and/or had lower occupational skill levels. For all other variables there was no significant difference (*p* > 0.05) between responders and non-responders (data not shown).

Within the compensable cohort (*n* = 179) that is, those who made a claim, rates of claim acceptance and legal representation at 6 months were investigated. These results are shown in Table 1. A CTP claim was made by 117 participants, with 80% being legally represented at 6 months compared to 48% of the 54 WC claimants. Only 55% of CTP claimants knew their claim was accepted compared to 82% of WC claimants. There were eight other claims (i.e. not CTP or WC). These differences were significant (*p* < 0.001) across the three groups (i.e. CTP, WC and other).

**Table 1:** Relationships^#^ between claim type and legal representation^a^ and claim acceptance among 179 participants who made a claim within first 6 months

	Claim type at 6 months		
	Compulsory Third Party No. (%)	Workers Compensation No. (%)	Other^b^ No. (%)
Legal representation at 6 months			
Yes	93 (79.5)	26 (48.1)	6 (75.0)
No	24 (20.5)	28 (51.9)	2 (25.0)
Claim acceptance at 6 months			
Yes	64 (54.7)	44 (81.5)	2 (25.0)
No	10 (8.5)	0 (0)	2 (25.0)
Don’t know	43 (36.8)	10 (18.5)	4 (50.0)

^a^Not all participants who sought legal representation made a claim (*n* = 3)

^b^(medical negligence = 1, Australian Defence Force (ADF) = 1, Department of Veteran Affairs (DVA) = 1, income protection insurance = 1, civil action = 1, not stated = 3)

^#^Relationships were considered significant for any association with *p* value <0.05

### Predictors of making a claim

In the unadjusted analysis the most significant predictors of making a claim reflected greater eligibility to claim such that participants who self-reported at fault were much less likely to claim (OR 0.14, 95%CI 0.08-0.23, *p* < 0.001), and participants who were involved in a crash on a public road were more likely to claim (OR 3.74, 95% CI 1.63-8.59, *p* = 0.002). For our study, we were interested in factors other than eligibility and the final model did not include these two variables; these results are shown in Table 2.

**Table 2:** Predictors for making a claim at 6 months (*n* = 294, 179 [61%] made a claim)

Variable^a^	Unadjusted OR (95% CI)	*P*	Adjusted^b^ OR (95% CI)	*P*
Body Mass Index (BMI)^e^ (kg/m^2^)		0.07		0.005
< 18.50 (underweight)	1.28 (0.27, 6.02)		0.87 (0.17, 4.40)	
18.50-24.99 (normal)	1.00		1.00	
≥ 25.00 (overweight)	2.14 (1.22, 3.76)		3.05 (1.63, 5.68)	
≥ 30.00(obese)	1.47 (0.80, 2.71)		1.63 (0.83, 3.20)	
Vehicle type		0.02		0.03
Motor vehicle	1.00		1.00	
Motorcycle	0.49 (0.30, 0.80)		0.47 (0.28, 0.82)	
Bicycle	0.66 (0.20, 2.16)		0.91 (0.26, 3.21)	
Index of Relative Socioeconomic Disadvantage		0.09		0.04
Most disadvantaged (quintile 1)	0.87 (0.42, 1.81)		0.70 (0.32, 1.55)	
More disadvantaged (quintile 2)	1.30 (0.44, 3.89)		1.13 (0.35, 3.67)	
Average (quintile 3)	1.00		1.00	
Less disadvantaged (quintile 4)	0.45 (0.22, 0.93)		0.37 (0.17, 0.82)	
Least disadvantaged (quintile 5)	0.62 (0.29, 1.33)		0.39 (0.17, 0.90)	
Male	0.56 (0.32, 0.95)	0.03		
Education skill level^c^		0.11		
Bachelor degree and above	1.00			
Certificate and advanced diploma	0.59 (0.30, 1.16)			
Secondary education	0.73 (0.37, 1.47)			
Pre-primary and primary education	5.19 (0.62, 43.6)			
Work hours before injury		0.07		
Fulltime	1.00			
Part time	2.44 (1.13, 5.25)			
Didn’t work	1.28 (0.72, 2.27)			
Language other than English (yes)	1.75 (1.05, 2.89)	0.03		
Risk of short term harm due to alcohol consumption (yes)	0.58 (0.36, 0.94)	0.03	0.56 (0.32, 0.97)	0.04
Pre-morbid neck pain in last 6 months (yes)	0.41 (0.14, 1.18)	0.10		
Self-assessed pre-injury health status^d^		0.08		0.05
Excellent	1.00		1.00	
Very good	1.12 (0.63, 1.98)		1.45 (0.77, 2.74)	
Good	1.13 (0.59, 2.14)		1.16 (0.58, 2.34)	
Fair-Poor	0.29 (0.10, 0.84)		0.30 (0.09, 0.94)	

^a^All variables with *p* value <0.20 (unadjusted) and *p* value ≤0.10 (adjusted) were included in the data analysis

^b^Adjusted for other variables in the column

^c^The measure for education is from the Australian Standard Classification of Education (ASCED), Cat. No. 1272.0, Australian Bureau of Statistics 2001

^d^Self-assessed pre-injury health status is based on Question 1 from the Short Form 36, version 2, (SF36v2)

^e^BMI classification is from the Global Database on Body Mass Index, World Health Organisation

The sensitivity analysis that investigated the impact of those eligibility variables (self-reported not at fault and crash on a public road) showed that BMI was the only significant predictor in the smaller eligible only model. All other variables were not significant. The effect sizes of the non-significant variables were all in the same direction with similar (but reduced) magnitudes, when compared to the full group. The overall performance of the models in two groups was similar with concordance index of 0.71 and 0.72 for the full model and smaller eligible model, respectively. The significant predictor of claiming compensation in the adjusted analysis was BMI. Obese or overweight participants were more likely to make a claim than those with normal or low BMI. Participants involved in a motorcycle crash and those at risk for short term harm (injury) due to alcohol consumption were less likely to make a claim than those who were not at risk. Those who were socioeconomically less disadvantaged and least disadvantaged were less likely to claim compared with those of average disadvantage and those with fair-poor health compared to those with excellent health were also less likely to claim. The C-statistic for the multivariable logistic regression model was 0.71 indicating that the predictive value of the model was acceptable. Values of 0.8-0.9 are considered excellent but higher values are rare [50].

### Predictors of seeking legal representation

As previously described, the final model did not include the two variables pertaining to eligibility. The significant predictors of self-reported fault (OR 0.08, 95%CI 0.03-0.19, *p* < 0.001) and crash on a public road (OR 9.16, 95% CI 1.84-45.7, *p* = 0.007) were removed from the analysis.

Participants who spoke a language other than English at home were more likely to seek legal representation, and those participants with a household income of ≤ AUD$39,999 were more likely to seek legal representation compared to participants with higher household income. Participants who were least socioeconomically disadvantaged were less likely to seek legal representation compared to participants with average disadvantage, this relationship was not linear. These results are displayed in Table 3. The C-statistic for the multivariable logistic regression model was 0.77.

**Table 3:** Predictors for seeking legal representation at 6 months (*n* = 179, 125 [70%] sought legal representation)

Variable^a^	Unadjusted OR (95% CI)	*P*	Adjusted^b^ OR (95% CI)	*P*
Language other than English (yes)	2.47 (1.23, 5.00)	0.01	2.80 (1.2, 6.52)	0.02
Index of Relative Socioeconomic Disadvantage		0.11		0.02
Most disadvantaged (quintile 1)	0.45 (0.16, 1.28)		0.35 (0.11, 1.07)	
More disadvantaged (quintile 2)	0.63 (0.15, 2.61)		0.98 (0.16, 5.86)	
Average (quintile 3)	1.00		1.00	
Less disadvantaged (quintile 4)	0.40 (0.13, 1.23)		0.50 (0.16, 1.60)	
Least disadvantaged (quintile 5)	0.23 (0.08, 0.69)		0.15 (0.04, 0.50)	
Male	0.47 (0.23, 0.99)	0.05		
Marital status		0.07		
Single	1.00			
Married/de facto	0.68 (0.34, 1.37)			
Divorced/widowed/separated	6.48 (0.80, 52.7)			
Work status before injury (working)	0.35 (0.14, 0.89)	0.03		
Recovery expectations for work (yes)	0.29 (0.06, 1.33)	0.11		
Total yearly household income (before tax, AUD) excluding number of people in household^±^		0.04		0.045
≤ $39,999	3.49 (1.29, 9.43)		3.63 (1.22, 10.72)	
$40,000-$79,999	1.10 (0.53, 2.32)		0.98 (0.44, 2.17)	
≥ $80,000	1.00		1.00	
Alcohol use in the past year		0.05		
Never	4.40 (1.15, 16.9)			
≤ 1/month	0.80 (0.28, 2.28)			
2-4 times/month	1.50 (0.50, 4.51)			
2-3 times/week	0.78 (0.26, 2.37)			
≥ 4 times/week	1.00			
Pre-morbid neck pain in last 6 months (yes)	0.20 (0.04, 1.14)	0.07		
Post-morbid neck pain (yes)	2.21 (0.91, 5.40)	0.08		

^a^All variables with *p* value <0.20 (unadjusted) and *p* value ≤0.10 (adjusted) were included in the data analysis

^b^Adjusted for other variables in the column

^±^Categories of income are from the Household, Income and Labour Dynamics in Australia (HILDA) Survey Wave 6 Household Questionnaire

To check for multicollinearity between the variables in the models, correlations and VIF were tested. The correlations found were small, that is: *r*_*s*_ < 0.4 and unlikely to cause any multicollinearity problems. This was confirmed with multicollinearity diagnostic testing using VIF with results showing VIFs ≤ 2 for all the variables. A VIF of greater than 5 or 10 usually indicates a multicollinearity problem.

## Discussion

In summary, the significant predictor of making a claim was being overweight or obese. Motorcycle crash, risk of short term harm due to alcohol consumption and poorer pre-injury health were associated with a decreased likelihood of making a claim. Amongst compensable participants, the predictors of seeking legal representation were largely related to socio-economic factors. Lastly, the differences between compensable and non-compensable participants were not related to physical pre-existing/baseline health status measures.

### Compensation status

It has been suggested that people with poorer health are more likely to claim than those in good health and that pre-injury/baseline health accounts for a poorer recovery not ‘exposure’ to compensation [10, 11]. We found that the differences between those ‘exposed’ (i.e. made a claim) and those ‘unexposed’ were not related to certain health measures. Bias from reverse causality was not detected in this cohort [12]. However, these results should be interpreted with caution due to the limited psychological variables measured.

The timeframes for claim acceptance reflect scheme design – WC is no-fault, CTP is fault-based, the latter can delay liability determinations. Likewise, explanations for high legal representation include liability issues, access to financial entitlements and/or the complexity of negotiating the claims process as reported previously [15, 23].

### Predictors of making a claim and seeking legal representation

Eligibility contributes to propensity to claim, which is as expected. However, injury severity (ISS/NISS) was not a predictor, which is unexpected given the moderate to severe injuries sustained by participants. It has been shown that those with minor injuries are far less likely to claim due to the inconvenience and effort required particularly if the injury does not cause any significant impact (e.g. minimal pain or loss of function) [18, 19]. For other factors, higher BMI has been associated with poorer physical and mental health, long term disability, and chronic pain [51–53]. In Australia, where almost 63% of adults are overweight or obese, obesity is a national health priority area and a significant public health problem [40]. Overweight or obese people could be faced with a prolonged recovery and therefore, more likely to claim. There is evidence linking obesity to increased WC claim rates and costs, and additional sick leave across numerous jurisdictions particularly for upper and lower limb injuries [54–56]. It follows that people with a higher BMI may be more likely to experience greater levels of disability and people with greater disability are more likely to make a claim.

For those less likely to claim, motorcyclists are more likely to be involved in single vehicle crashes [57]. In these crashes there is no one to claim against [31]. In addition, speeding and alcohol are stronger contributors to single vehicle crashes, which could result in traffic and/or criminal violations [57]. In NSW, a police report is required to make a claim and motorcyclists could be less likely to approach police under these circumstances [58, 59]. These are plausible reasons for the low claim rate in motorcyclists.

Similarly, short term harm due to greater alcohol consumption increases the risk of alcohol-related injury [60]. The most common cause of death due to intoxication is a road traffic crash [61], and alcohol consumption is linked to numerous medical conditions, which along with pre-injury fair-poor health could be associated with being more or most disadvantaged and/or not understanding how to claim. This last comment should be interpreted cautiously due to small numbers in this group (31/452, 7%). In addition, those who are socioeconomically less or least disadvantaged are more likely to have higher levels of education and be employed in professional and/or associated professional jobs (e.g. managers and administrators), and less likely to have co-morbidities. This is likely to reduce their need to claim for economic and other losses such as medical expenses [62].

Besides eligibility, a person’s decision whether or not to make a claim can also be influenced by other factors. These include a perception their injury is too minor, concerns about current or future employment options, and/or a lack of knowledge about eligibility to claim [18, 19]. Our study population sustained moderate to severe, not minor injuries, so this is unlikely to have been a factor. Concerns about current or future employment options and a lack of knowledge about eligibility to claim are possible but they were not measured in this study.

The sensitivity analysis for only those eligible to make a claim, showed that higher BMI was the one significant predictor in this smaller model, but the reduced effect sizes of all the other variables (i.e. those that were significant in the larger model) could indicate that these variables may be less relevant when restricted to the eligible population and/or a larger sample size is needed in future research.

The predictors of legal representation were speaking a language other than English at home and a low household income. These factors are commonly associated with health inequities (e.g. increased illness and disability, poor access to health services, and poor health literacy) [62, 63]. These inequities could lead to increased legal representation due to the complexity of managing a claim (e.g. understanding legal terminology to access financial entitlements in the circumstances surrounding the crash and fault status) and/or accessing health care services via a third party payer (the insurer) [15, 23]. Alternatively, people who are less or least socioeconomically disadvantaged may not require legal assistance to access financial entitlements because of greater competency navigating the claims process particularly if work capacity is not affected.

Moreover, qualitative research shows people feel they require legal representation to assist with adversarial claims processes, accessing reasonable entitlements, perceived illegitimacy of injury, and system disorganisation (e.g. communication and administrative deficits) [21, 23, 24]. It is feasible these factors would be challenging to people with limited English proficiency and those from lower socio-economic backgrounds particularly in the presence of physical or psychological limitations.

### Strengths and limitations

Our prospective study was a trauma cohort of moderate to severe injuries involving upper and lower limb fractures. We used validated and standardised measures. Participants were predominantly male, from lower socio-economic backgrounds with a household income below AUD $80,000. Although reflective of a more severe trauma population, they may not be representative of all CTP and WC claimants. The issues surrounding eligibility to claim are complex, dependent on scheme design and involve a myriad of legal interpretations.

It would have been beneficial to measure self-reported fault by including the constructs of blame, perceived injustice and/or attributions of responsibility [64, 65]. Recent research has shown that these factors are significant predictors of poorer health outcomes [5, 64, 65]. Fault (i.e. the driver caused the crash) is not the same as blame (i.e. blaming someone or something for the injury) [64, 65]. For example, a driver may have ‘caused’ the crash, but blame his/her passenger for distracting them or poor road conditions. Blame or perceived injustice do not necessarily mean access to compensation. Our singular measure did not encompass these constructs.

Further, the collection of baseline psychological variables would have been useful (e.g. depression, pain catastrophising and/or anxiety). Poor baseline mental health and stressfulness has been associated with poor recovery in a compensable setting; which could impact on making a claim and seeking legal representation [14, 15]. Other limitations were recruitment of participants solely from hospital and moderate loss to follow-up (32%).

Lastly, we did not include any indices of social support. There is growing awareness of the importance of social support to aid injury recovery and return to work [66]. There are a number of validated measures of workplace and family support and future research would benefit from their inclusion [67, 68].

### Future research and policy implications

The predictors of making a claim illustrate the problems associated with a higher BMI and how this extends into the compensable arena. However, scheme regulators and insurers are limited in their capacity to address this significant societal issue. Conversely, those less likely to claim may benefit from access to health care services and financial entitlements and, if socioeconomically less or least disadvantaged, may have no need to claim for these items. The predictors of seeking legal representation provide insight into the importance of socio-economic and language factors.

Given the limited research, these factors need to be explored in different populations with alternative compensation systems to determine whether the findings are replicable. The presence of reverse causality bias should be routinely investigated if compensation related factors are potential confounders.

For policy makers there is an opportunity to conduct risk assessments, identify those likely to struggle post injury, and attempt to mitigate that risk with proactive health interventions and claims management. In addition, extra assistance for claimants from CALD and lower socio-economic backgrounds may alleviate some of the pressure to seek external advice. For example: face-face meetings conducted in an appropriate language; a streamlined claims process; and/or early payments for treatment and financial hardship. Conversely, those socioeconomically less or least disadvantaged may benefit from minimal insurer intervention.

Finally, the generalisability of our results could be affected by the diverse and complex socio-political environment of compensation schemes. For example, NSW has a predominantly fault-based modified common law CTP scheme; whereas other Australian states have purely common law or no-fault CTP schemes. Internationally, compensation schemes are based on mechanism of injury and/or type of disability, and/or governing legislation to access financial entitlements. Notwithstanding that, themes from qualitative research appear to be consistent across jurisdictions and countries [21, 22]. Further, increased BMI has been associated with greater absenteeism, healthcare costs and claim rates across numerous jurisdictions, albeit in larger cohorts [55, 56].

## Conclusion

Seeking financial compensation was associated with a higher pre-injury BMI rather than injury-related factors. Seeking legal representation was solely related to socio-economic factors. Evidence to date suggests these relationships are complex, population specific and dependent on scheme design.

## Abbreviations

ABS: Australian Bureau of Statistics
ADF: Australian Defence Force
AIS: Abbreviated injury scale
ANF: Accident notification form
ANOVA: Analysis of variance
ASCED: Australian Standard Classification of Education
ASCO: Australian Standard Classification of Occupations
AUD: Australian dollar
AUDIT-C: Alcohol use disorders identification test: self-report version
BEACH: Bettering the evaluation of care and health
BMI: Body mass index
CALD: Culturally and linguistically diverse
CI: Confidence interval
CTP: Compulsory third party
DVA: Department of Veteran Affairs
GCS: Glasgow coma scale
HILDA: Household, income and labour dynamics in Australia
IRSD: Index of relative socioeconomic disadvantage
ISS: Injury severity score
MAA: Motor accidents authority
NHMRC: National Health and Medical Research Council
NISS: New injury severity score
NSW: New South Wales
OR: Odds ratio
PCS: Physical component score
SD: Standard deviation
SF36v2: Short form-36 version 2.0
SIRA: State insurance regulatory authority
VIF: Variance inflation factor
WC: Workers compensation
